# Obstructive Sleep Apnea in Patients with Head and Neck Cancer—More than Just a Comorbidity?

**DOI:** 10.3390/medicina57111174

**Published:** 2021-10-28

**Authors:** Christopher Seifen, Tilman Huppertz, Christoph Matthias, Haralampos Gouveris

**Affiliations:** Hals-, Nasen-, Ohrenklinik und Poliklinik, Universitätsmedizin der Johann Gutenberg—Universität Mainz, 55131 Mainz, Germany; tilman.huppertz@unimedizin-mainz.de (T.H.); christoph.matthias@unimedizin-mainz.de (C.M.); haralampos.gouveris@unimedizin-mainz.de (H.G.)

**Keywords:** obstructive sleep apnea, head and neck cancer, comorbidity, outcome, review

## Abstract

Obstructive sleep apnea is the most common type of sleep-disordered breathing with growing prevalence. Its presence has been associated with poor quality of life and serious comorbidities. There is increasing evidence for coexisting obstructive sleep apnea in patients suffering from head and neck cancer, a condition that ranks among the top ten most common types of cancer worldwide. Routinely, patients with head and neck cancer are treated with surgery, radiation therapy, chemotherapy, immunotherapy or a combination of these, all possibly interfering with the anatomy of the oral cavity, pharynx or larynx. Thus, cancer treatment might worsen already existing obstructive sleep apnea or trigger its occurrence. Hypoxia, the hallmark feature of obstructive sleep apnea, has an impact on cancer biology and its cure. Early diagnosis and sufficient treatment of coexisting obstructive sleep apnea in patients with head and neck cancer may improve quality of life and could also potentially improve oncological outcomes.

## 1. Introduction

Obstructive sleep apnea (OSA) is the most common type of sleep-disordered breathing, with a prevalence ranging between 4% and 24% in middle-aged people [1]. In addition, the prevalence of sleep apnea has been reported to be 49.7% in men and 23.4% in women aged 40 years or older in a large population-based sample in Western Europe [2]. OSA is anatomically defined by the difficulty of keeping the patent upper airway during sleep, resulting in partial or complete airway obstruction, sleep disruption and leading to the clinical picture of daytime sleepiness or fatigue and a missing sense of recovery after sleep [3]. Furthermore, OSA has been related to increased risk for cardio and cerebrovascular events, development of metabolic syndrome, kidney diseases, chronic obstructive pulmonary disease (COPD) and asthma [4,5,6,7,8,9,10,11]. There is evidence of increased cancer mortality in patients with OSA during follow-up compared to general population samples [12,13]. Different authors reported coexisting OSA in patients with head and neck cancer, especially after therapy [14,15,16,17,18,19]. In addition, recent evidence suggests very high OSA prevalence in patients with head and neck cancer even pre-treatment [16]. Head and neck cancer ranks among the top ten most common types of cancer worldwide with a severe impact on quality of life and elevated mortality in affected individuals [20,21]. Although there appears to be an elevated OSA incidence in patients with head and neck cancer, this comorbidity and particularly its possible linkage has been poorly studied in small prospective studies [22].

In this review, we describe the comorbidity of OSA in patients with head and neck cancer. We highlight the latest studies on the subject. We speculate about whether OSA and head and neck cancer might be more than just comorbid. Finally, we suggest what focus future research should have in order to close open knowledge gaps on this issue.

For this narrative review, a search of the literature up to 1 July 2021 was performed by using MEDLINE on terms including “sleep apnea and head squamous cell carcinoma” (HNSCC), “sleep apnea and HNSCC”, “obstructive sleep apnea and head and neck squamous cell carcinoma”, “OSA and head and neck squamous cell carcinoma”, “obstructive sleep apnea and HNSCC” and “OSA and HNSCC”. Considered for inclusion were both prospective as well as retrospective studies that included Apnea-Hypopnea Index (AHI) as part of their primary or secondary outcomes in patients with head and neck squamous cell carcinoma.

## 2. Obstructive Sleep Apnea and Cancer Risk

The clinical picture of OSA was first described by William Osler in 1918 [23]. OSA is characterized by recurrent episodes of partial or complete airway obstruction during sleep leading to repetitive apneas or hypopneas. Airway obstruction results from upper airway collapse or anatomic airway obstruction, even though respiratory effort is still present [3]. Being overweight, and in particular elevated BMI, is the strongest risk factor for OSA [24]. Accordingly, a large neck circumference is also associated with an increased risk for OSA [25]. Furthermore, OSA prevalence increases with growing age and is more frequently seen in males [26,27]. The diagnosis of OSA is often delayed because individuals are rarely aware of their sleep disorder [28]. Clinical symptoms are manifold: Most commonly, patients complain about excessive daytime sleepiness or daytime fatigue, dry or sore throat, gastroesophageal reflux or sexual dysfunction, to name but a few [3,29]. Notably, head and neck cancer can mimic OSA by causing typical OSA symptoms. Head and neck cancer should be considered in patients experiencing symptoms besides typical symptoms of OSA and particularly in patients with lower BMI. A scoping review of Moore and colleagues summarized 79 cases of OSA being the leading symptom of head and neck cancer [30]. The authors suggested flexible nasal endoscopy as a potential tool for OSA evaluation in selected individuals since the causative malignant pathology was visible in all examined patients [30].

The diagnosis of OSA is based on clinical symptoms, the existence of risk factors and sleep study evaluation (polysomnography, a portable home-based test). In order to assess the severity of sleep apnea, the Apnea-Hypopnea Index (AHI) is used. The AHI is based on the total number of apneas (cessation in airflow ≥10 s) and hypopneas (≥30% reduction in the flow of air associated with ≥4% desaturation in O2 level) occurring per hour of sleep. An AHI of 5–15 is classified as mild, 15–30 is considered moderate and greater than 30 is considered severe [31]. The treatment of mild OSA involves behavioral or lifestyle modification, including loss of weight and avoiding alcohol. The first-line therapy for moderate to severe sleep apnea is the use of positive airway pressure (PAP) to keep an individual’s airway open during sleep [32,33]. Although the efficacy of PAP is high, 46–83% of patients are non-adherent to therapy [34]. For moderate OSA, there are multiple surgical treatment options, e.g., palatal, skeletal, tonsillar or tongue surgeries, hypoglossal nerve stimulation and mandibular advancement devices, while palatal surgeries (uvulopalatopharyngoplasty, laser-assisted uvuloplasty) are most often performed [35]. For severe OSA in patients intolerant to PAP, hypoglossal nerve stimulation is currently the only reliable alternative to PAP therapy [36].

The role of comorbidities in OSA patients has emerged over the years, and new conditions are increasingly reported. The best-studied comorbidity in OSA is systemic hypertension [8]. Patients with severe OSA were reported to have a high cardiovascular risk, which was normalized by CPAP treatment [7]. Likewise, studies reported an increased risk of strokes in OSA patients [6]. The metabolic syndrome is highly prevalent in OSA patients, and OSA might play a role in the pathogenesis of insulin resistance [11,37]. Furthermore, there is evidence that kidney function worsens due to OSA; however, OSA and renal diseases share common risk factors [4]. The association of COPD and OSA is known as “overlap syndrome”, whose prevalence was found to increase with age [9]. OSA is more common in asthmatics than in controls, and OSA was associated with a higher frequency of asthma exacerbations [10]. Additionally, OSA is associated with glaucoma, a highly prevalent disorder [38].

The association of OSA and cancer is of special interest, and its exploration started throughout the last few years. Broad epidemiological studies demonstrated increased cancer mortality of patients with coexisting OSA compared to general population samples during follow-up [12,13]. Nieto and colleagues were among the first to demonstrate that OSA was associated with total and cancer mortality in a dose-response fashion in a community-based sample [13]. Furthermore, a large multicenter cohort study by Justeau and colleagues found that nocturnal hypoxemia predicted all-cancer incidence in patients investigated for suspected OSA, independently of major confounding factors [39]. Older people with obesity and no adequate OSA therapy were most often affected, and the most common types of cancer were lung and breast [39]. Li and colleagues described OSA severity as a risk factor that contributed to short overall survival in patients with lung cancer [6]. A study by Huppertz and colleagues demonstrated a significant association between recurrent disease or cancer-related mortality and higher AHI in patients with head and neck squamous cell carcinoma (HNSCC) [16].

## 3. Obstructive Sleep Apnea Might Be a Relevant Comorbidity of Head and Neck Cancer

Regarding HNSCC, different authors reported coexisting OSA [14,15,16,17,18,19,40,41,42,43,44,45]. Worldwide, over 500,000 new cases of HNSCC are reported annually, being the seventh most common type of cancer [20,46]. HNSCC occurs within the oral cavity, pharynx or larynx. Tobacco and alcohol consumption are well-established primary risk factors for the development of HNSCC, with synergistic effects [21]. Over the last few years, there has been a shift towards a steady increase in oropharyngeal squamous cell carcinoma (OPSCC) and a steady decrease in laryngeal and hypopharyngeal squamous cell carcinoma. A parallel decrease in smoking and an increased exposure to oncogenic human papillomavirus (HPV) as primary risk factors for the development of OPSCC has been observed [20,47]. Symptoms of HNSCC are manifold, and treatment is stage-dependent, involving surgery, radiation therapy, chemotherapy, immunotherapy or a combination of these. The interested reader is referred to more profound reviews on this topic as further details are beyond the scope of the present review [20,21].

There is evidence of coexisting OSA in patients with head and neck cancer, yet its diagnosis and treatment have not been extensively studied: In 1980, Zorick and colleagues firstly described coexisting OSA in a patient with lymphocytic lymphoma in the head and neck region, with OSA being the presenting symptom that subsequently improved after therapy [48]. In 2005, Payne and colleagues found that pre-treatment OSA prevalence was 76% in patients with malignancies of the oral cavity or oropharynx. Patients with an AHI ≥20 had a tendency to have increased postoperative complications [18]. Huppertz and colleagues showed an OSA prevalence of 90% in patients with HNSCC pre-treatment, increasing to 94% post-treatment [16]. Accordingly, Quyang and colleagues showed that OSA prevalence increased from 50% to 82.5% when laryngeal cancer was treated with laryngeal function preservation surgery [44]. Comparing their data, they found that AHI was significantly higher in patients treated with supracricoid partial laryngectomy than in patients treated with vertical partial laryngectomy [44]. A study by Friedman and colleagues found an OSA incidence of 91.7% after successful treatment of a mixed group of patients with head and neck cancer [15]. Published in 2001, this group was the first to highlight the occurrence of OSA in patients with head and neck cancer [15]. Two independent studies from Israel and Teixeira and colleagues showed that OSA incidence or prevalence after surgical treatment of laryngeal cancer was 81–91% and 92.3%, respectively [42,45]. Among other findings, Israel and colleagues reported the presence of OSA was 81% in patients treated with supracricoid horizontal partial laryngectomy and 91% in patients treated with frontolateral vertical partial laryngectomy [42]. Teixeira and colleagues compared the severity of OSA in patients submitted to horizontal and vertical partial laryngectomy and found that AHI was 36.9/h in patients submitted to partial vertical laryngectomy and 11.2/h in individuals offered partial horizontal laryngectomy [45]. Faiz and colleagues reported an OSA prevalence of 88% after radiation therapy in patients with head and neck cancer [14]. Contrarily, Nesse and colleagues found an OSA prevalence of only 12% in patients followed up between 6 months and 5 years after treatment of malignancies of the oral cavity or oropharynx [17]. Steffen and colleagues reported an OSA prevalence of 19% in patients following treatment of HNSCC, while radiation therapy had no impact on prevalence [19]. Further studies on the subject are summarized in Table 1.

## 4. Discussion

In summary, the aforementioned studies show that OSA is far more prevalent in patients with head and neck cancer compared to the general population. OSA, as well as HNSCC, causes poor quality of life. In a systematic review of 10 prospective studies on the subject, OSA incidence ranged from 12% to 95.8%, with a weighted average of 59.78% on patients treated for head and neck cancer [22].

These numbers show how controversial the literature still is on the incidence of OSA in patients with HNSCC. However, most studies evaluated OSA prevalence in patients with HNSCC after treatment, as portable home sleep apnea testing or polysomnography is not included in standard pre-treatment diagnostics. The treatment of head and neck cancer, primarily surgery and radiation therapy, alters the anatomy of the oral cavity, pharynx or larynx. For instance, patients treated with partial laryngectomy were predisposed to OSA due to structural alterations [44]. Other investigators suggested that post-radiation edema in patients with head and neck cancer might be a risk factor for OSA [41]; they observed that patients with OSA had a shorter time interval between the conclusion of radiation therapy and the sleep study date [41]. Pre-treatment occurrence of OSA in patients with head and neck cancer can be explained by structural anomalies due to growing masses with airway obstruction. When focusing on post-treatment occurrence of OSA, structural alterations through surgery and/or radiation therapy can once again be causal. Another potential explanation of the comorbidity could be the involvement and dysfunction of various cranial nerves, e.g., anatomical impairment of the nerves by lymph node metastases and/or the primary tumor itself or through neuropathies induced by radiation therapy and/or chemotherapy. While the linkage between the occurrence of OSA in patients with head and neck cancer can be plausibly explained, it remains unclear if OSA contributes to the development and progression of head and neck cancer. To end this, it is essential to develop a more detailed insight at the molecular level.

Detailed pathophysiological mechanisms are still controversial. The present evidence exists on the basis of clinical data that are presented in this review. Further deciphering of molecular or cellular mechanisms is still missing and a matter of future investigations. Recent research mainly has focused on OSA incidence or prevalence in patients with head and neck cancer and the impact of different therapy regimens on these parameters. On a molecular level, the linkage between OSA-related intermittent hypoxia and HNSCC tumor progression is not well understood. Intermittent hypoxia is the pathophysiological hallmark feature of OSA. Hypoxia is established as one of the most important causes of resistance to radiation therapy and chemotherapy [49,50]. Thus, high AHI resulting in serious intermittent hypoxia might contribute to the failure of radiation therapy or chemotherapy, which in turn might increase the rate of cancer recurrence or cancer-related mortality. On a molecular level, hypoxia induces the transcription of Hypoxia-inducible factor-1α (HIF-1α), a key molecule that promotes an aggressive tumor phenotype, by stimulating angiogenesis, tissue invasion or metastasis [49]. Intermittent hypoxia exposure has been associated with accelerated tumor growth progression, metastases and therapy resistance in a murine model of subcutaneous melanoma [4]. Albeit carcinogenesis is of different origin, OSA-related intermittent hypoxia appears to have nourishing effects on tumor progression. Similarly, OSA has been associated with systemic inflammation and with classic pro-inflammatory cytokines, such as Interleukin 6 (IL-6) or Tumor necrosis factor-α (TNF-α), both promoting a persistent low-intensity inflammatory state [51,52,53]. In turn, inflammation substantially contributes to the development and progression of tumors [54]. Conversely, one could hypothesize that sufficient treatment of coexisting OSA in patients with HNSCC would optimize the therapy regimen and thus could potentially contribute to better HNSCC-related primary oncologic outcomes and possibly reduce the risk of HNSCC recurrence.

Currently, OSA screening in patients diagnosed with head and neck cancer is not an established clinical practice. As numerous studies highlight OSA as a relevant comorbidity in patients with head and neck cancer, its diagnosis should not be missed. However, once the diagnosis of HNSCC has been made, its definitive treatment certainly must be carried out promptly, leaving limited time for OSA screening. When pre-treatment OSA screening is not possible, it should be reconsidered after completion of definitive treatment, e.g., as part of regular follow-up care. It is imperative that health care professionals understand the far-reaching and profound effects of OSA, particularly in patients with HNSCC. Selected patients could benefit from early diagnosis and sufficient treatment of coexisting OSA, although patients with HNSCC and comorbid OSA may be more difficult to treat. Additionally, these measures could be cost-effective tools to reduce long-term morbidity and mortality. Studies to test whether OSA treatment in HNSCC patients could improve oncologic outcomes are still missing. Hence, future studies should investigate the specific impact of sufficient treatment of coexisting OSA in patients with head and neck cancer. Moreover, given the extremely high prevalence of OSA in HNSCC patients, it is still unknown whether accompanying fatigue results from head and neck cancer itself or comorbid OSA in these patients. It is conceivable that early diagnosis and sufficient therapy of OSA in patients with head and neck cancer could lead to the improved oncologic outcomes and increased quality of life, thus optimizing the fight against HNSCC.

## 5. Conclusions

An increasing number of studies showed that OSA is far more prevalent in patients with head and neck cancer compared to the general population. However, a possible linkage between OSA and head and neck cancer remains unclear.

## Figures and Tables

**Table 1:** Studies on the comorbidity of obstructive sleep apnea and head and neck cancer.

Author and Year of Publication	Patients Included (*n*)	Study Design	Cancer Localization	OSA Incidence Pre- (Pre) and/or Post-Treatment (Post)	Treatment Method
Friedman et al., 2001	24	Prospective cohort	Soft palate, larynx, supraglottic larynx, tongue base, pharynx	91.7% post	Radiation therapy; Other than radiation therapy
Payne et al., 2005	17	Prospective cohort	Oral cavity, oropharynx	76% pre	
Israel et al., 2006	22	Prospective cohort	Larynx	81–91% post	Surgery
Nesse et al., 2006	33	Prospective cohort	Oral cavity, oropharynx	12% post	Surgery; Surgery and radiation therapy; Radiation therapy
Steffen et al., 2009	31	Prospective cohort	Oropharynx, larynx	19% post	Surgery; Surgery and radiation therapy
Teixeira et al., 2013	14	Prospective cohort	Larynx	92.3% post	Surgery
Faiz et al., 2014	56	Retrospective cohort	Mucosa, skin, salivary gland, primary neck involvement	67% pre, 88% post	Radiation therapy; Other
Huyett et al., 2017	16	Prospective cohort	Oropharynx, larynx	50% post	Radiation therapy
Loth et al., 2017	51	Prospective cohort	Oropharynx	25.49% post	Radio-chemotherapy; Surgery and Radio-chemotherapy
Quyang et al., 2019	40	Prospective cohort	Larynx	57.5% pre, 82.5% post	Surgery
Huppertz et al., 2020	33	Prospective cohort	Tongue, oropharynx, hypopharynx	90% pre, 94% post	Radio-chemotherapy; Surgery and radiation therapy

## Data Availability

Not applicable.
